# The effects of experimental knee pain on lower limb corticospinal and motor cortex excitability

**DOI:** 10.1186/s13075-015-0724-0

**Published:** 2015-08-12

**Authors:** David Andrew Rice, Thomas Graven-Nielsen, Gwyn Nancy Lewis, Peter John McNair, Nicola Dalbeth

**Affiliations:** Health and Rehabilitation Research Institute, Auckland University of Technology, 55 Wellesley Street East, Auckland, 1010 New Zealand; Waitemata Pain Services, Department of Anaesthesiology and Perioperative Medicine, Waitemata District Health Board, Shakespeare Road, Auckland, 0620 New Zealand; Center for Sensory-Motor Interaction (SMI), Department of Health Science and Technology, Faculty of Medicine, Aalborg University, Fredrik Bajers Vej 5, 9100 Aalborg, Denmark; Department of Medicine, University of Auckland, 2 Park Road, Auckland, 1023 New Zealand

## Abstract

**Introduction:**

Notable weakness of the quadriceps muscles is typically observed as a consequence of knee joint arthritis, knee surgery and knee injury. This is partly due to ongoing neural inhibition that prevents the central nervous system from fully activating the quadriceps, a process known as arthrogenic muscle inhibition (AMI). To investigate the mechanisms underlying AMI, this study explored the effects of experimental knee pain on lower limb corticospinal and motor cortex excitability.

**Methods:**

Twenty-four healthy volunteers participated in this study. In experiment 1, experimental knee pain was induced by the injection of hypertonic saline into the infrapatellar fat pad (n = 18). In experiment 2, isotonic saline was injected into the fat pad as a non-painful control (n = 8). Pain intensity was measured on a 10-cm electronic visual analogue scale. Transcranial magnetic stimulation and electromyography were used to measure lower limb motor-evoked potential amplitude and short-interval intracortical inhibition before and after the injection.

**Results:**

The peak VAS score following hypertonic saline (5.0 ± 0.5 cm) was higher than after isotonic saline (*p* <0.001). Compared with baseline, there was a significant increase in vastus lateralis (*p* = 0.02) and vastus medialis motor-evoked potential amplitude (*p* = 0.02) during experimental knee pain that was not apparent during the control condition. Biceps femoris and tibialis anterior motor-evoked potential amplitude did not change following injection (all *p* >0.05). There was no change in short-interval intracortical inhibition measured from vastus lateralis following injection (both *p* >0.05).

**Conclusions:**

Quadriceps corticospinal excitability increases during experimental knee pain, providing no evidence for a supraspinal contribution to quadriceps AMI.

## Introduction

Marked and sustained weakness of the quadriceps muscle is often observed in response to knee joint injury, surgery and pathology. This weakness is partly due to an ongoing muscle activation deficit characterised by an inability of supraspinal pathways to voluntarily drive the quadriceps muscle, known as arthrogenic muscle inhibition (AMI) (for a review see [1]). Quadriceps AMI is ubiquitous across a range of knee joint conditions including osteoarthritis (OA) [2], rheumatoid arthritis [3], anterior knee pain [4], patella contusion [5], after anterior cruciate ligament (ACL) injury [4] and reconstruction [4], after meniscus injury and repair [6] and following total knee joint arthroplasty [7].

As well as being a direct cause of quadriceps muscle weakness, AMI can prevent effective muscle strengthening [8, 9], leading to long-term quadriceps muscle weakness that is difficult to reverse. Ongoing quadriceps weakness is clinically important, as it is associated with impaired physical function [10–12] and knee joint instability [13]. Furthermore, quadriceps weakness may increase the rate of loading at the knee joint [14, 15] with recent longitudinal studies showing that greater baseline quadriceps strength may protect against incident knee pain [16, 17], patellofemoral cartilage loss [16] and tibiofemoral joint space narrowing [18].

Nociceptive output from the affected knee joint is thought to play an important role in mediating quadriceps weakness. Several studies have shown an association between knee pain intensity and the magnitude of quadriceps voluntary activation deficits [19, 20] and, in some instances, reducing knee pain has been shown to improve quadriceps strength [21, 22]. Recent studies using an experimental knee pain model based on hypertonic saline injections into the infrapatellar fat pad have demonstrated that acute knee pain leads to an immediate decrease in quadriceps peak torque during both isometric and isokinetic testing [23, 24]. Interestingly, hamstrings peak torque was also reduced in the presence of knee pain [23]. Furthermore, a significant association was observed between knee pain intensity and the subsequent change in muscle strength [23]. These findings show that nociception alone is sufficient to induce quadriceps AMI and suggest that articular nociception inhibits the activation of other muscles around the joint.

At least part of the inhibitory response in the quadriceps appears to be mediated at a spinal cord level, as experimental knee pain has been shown to inhibit quadriceps H-reflex amplitude [24], a measure of spinal reflex excitability that is strongly influenced by spinal α-motoneuronal excitability. Furthermore, animal studies have shown that nociceptive knee joint afferents synapse with spinal interneurons mediating group I non-reciprocal inhibition [25] and the flexion reflex [26], both of which produce a predominant pattern of extensor muscle inhibition.

Previous studies in the upper limb [27, 28] have shown that acute pain also leads to inhibition of motor pathways at the level of the motor cortex. Following experimental muscle pain the size of motor-evoked potentials (MEPs) has been shown to decrease significantly in response to transcranial magnetic stimulation (TMS) of the primary motor cortex [27, 28]. Part of this inhibition appears to occur at a cortical level, as depression of the MEP was shown to precede a decrease in H-reflex amplitude recorded from the same muscle [27]. Furthermore, Schabrun et al. [28] demonstrated recently that short-interval intracortical inhibition (SICI), a measure of GABAergic inhibition within the motor cortex, increases after experimental muscle pain.

It remains unknown whether acute knee pain leads to inhibition of lower limb muscles such as the quadriceps at the level of the motor cortex, or whether this inhibition occurs purely at a spinal reflex level. The aim of this study was to examine the effects of experimental knee pain on lower limb MEP amplitudes and quadriceps SICI. Our main hypotheses were that experimental knee pain would lead to a decrease in quadriceps MEP amplitude and an increase in SICI.

## Methods

### Participants

Healthy, pain-free participants volunteered to take part in this study, which consisted of two experiments. Experiment 1 included 18 participants (11 female, seven male) with a mean (± SD) age, height and weight of 29 ± 8 years, 1.72 ± 0.07 m, and 74 ± 14 kg, respectively. Experiment 2 included eight participants (six female, two male) with a mean (± SD) age, height, and weight of 22 ± 8 years, 1.69 ± 0.06 m, and 64 ± 13 kg, respectively. Four participants (three female, one male) were involved in both experiment 1 and experiment 2. All participants were screened and excluded based on contraindications to TMS, including epilepsy, head injury, metal implants, or central nervous system-altering medications. Further exclusion criteria were a previous history of knee joint pathology, lower limb or spinal surgery, neurological disease or a chronic pain condition. Participants were asked to refrain from ingesting caffeine, alcohol or medication 4 hours prior to testing. All participants provided written informed consent for the experimental procedures. Ethical approval for this study was granted by the Northern Y Regional Ethics Committee, Auckland, New Zealand (NTY 10-11-89) and conformed to the principles in the declaration of Helsinki.

### Experimental knee pain

Experimental knee pain was induced by injecting sterile hypertonic saline (5.8 %, 0.25 ml) into the right infrapatellar fat pad with the knee resting in slight flexion (experiment 1). Injections were performed with a 25-gauge needle mounted on a 1-ml syringe. Injections were from a medial approach with the needle inserted approximately 1 cm at a 45° angle in a posterolateral direction [29]. All injections were performed under sterile conditions. When injecting isotonic saline (0.9 %, 0.25 ml) as a control (experiment 2), the same procedures as above were used. Following needle withdrawal, participants continuously rated their knee pain on an electronic visual analogue scale (VAS) where 0 indicated “no pain” and 10 anchored “maximal pain”. The VAS data were sampled at 200 Hz.

### Transcranial magnetic stimulation

Lower limb MEPs were recorded using bipolar Ag-AgCl electromyography (EMG) electrodes (Norotrode 20, Myotronics Inc., Kent, WA, USA) with an interelectrode distance of 2.2 cm mounted on the skin overlying the vastus lateralis (VL), vastus medialis (VM), biceps femoris (BF), and tibialis anterior (TA) muscles of the right leg in accordance with established EMG guidelines [30]. A ground electrode (Red Dot, 3 M, St Paul, MN, USA) was positioned overlying the proximal tibia. Prior to electrode placement the skin was shaved, abraded and cleaned with alcohol to reduce signal impedance. All EMG signals were amplified (x1000), filtered (10–1000 Hz) (AMT-8, Bortec Biomedical Ltd., Calgary, AB, Canada) and sampled at 2000 Hz (Micro 1401, Cambridge Electronic Design, Cambridge, UK) before being stored on a computer for further analysis.

Transcranial magnetic stimuli were delivered over the scalp using a double cone coil (BiStim 200^2^, Magstim Company Ltd., Whitland, UK). The coil was placed over the contralateral (left) primary motor cortex so that the induced current flow was in a posterior-anterior direction. First, the optimum site for stimulation (hot spot) was found by delivering a series of suprathreshold stimuli as the coil was systematically moved over the scalp until the largest VL MEP was elicited. This was typically found approximately 1–2 cm lateral and anterior to the vertex. The hot spot was marked on the scalp with a felt pen and all further testing completed with the coil held directly over this position. The location of the coil on the participant’s head was visually checked throughout the experiment to ensure the location and angle of stimulation remained unchanged. Resting motor threshold (RMT) was determined using a staircase method and was defined as the lowest stimulation intensity (percentage of maximum stimulator output) evoking a MEP >50 μV in at least four of eight consecutive stimuli. Single-pulse TMS (test stimuli only) over the VL hotspot was used to elicit MEPs, while paired-pulse TMS (conditioning and test stimuli) was used to measure SICI. The test stimuli were delivered with an intensity sufficient to achieve a VL MEP amplitude that was approximately 5 % of the maximum M-wave recorded following electrical stimulation of the femoral nerve. As TMS over the lower limb representation typically elicits MEPs in a number of different muscles, MEPs were recorded in VL, VM, BF and TA muscles despite optimising the stimulus location and intensity for the VL muscle. SICI was measured from VL only. To ensure a thorough evaluation of SICI [31], three different intensities of conditioning stimuli (55 %, 65 % and 75 % of RMT) were used, with an interstimulus interval of 2 ms between the test and conditioning stimuli [32].

### Experimental protocols

#### Experiment 1: experimental knee pain

Measurements of lower limb MEP amplitude and VL SICI were performed before pain (prior to hypertonic saline injection), during pain (60 seconds after needle withdrawal), and after pain (when knee pain had returned to 0/10 on the VAS). In all conditions, ten single-pulse and 30 paired-pulse stimuli (ten stimuli for each of the three conditioning stimulus intensities) were delivered to evaluate SICI. The order of these stimuli was randomised. To evaluate changes in MEP amplitude during pain and after pain, ten additional single-pulse stimuli were delivered prior to the evaluation of SICI using the same test stimulus intensity as the before pain condition. If necessary, the test stimulus intensity was then adjusted for the subsequent measures of SICI to ensure that a test (unconditioned) MEP of the same size (±10 %) as the before pain test MEP was elicited [33, 34]. The conditioning stimulus intensities (55 %, 65 % and 75 % of RMT) remained unchanged across all measurement points, even if the test stimulus intensity was adjusted.

#### Experiment 2: control

Measurements of lower limb MEP amplitude and VL SICI were taken before and after the injection of isotonic saline into the infrapatellar fat pad, performed in an identical manner to the hypertonic saline injections. As isotonic saline does not usually induce pain, measurement time periods were matched to the average time of the during pain (60 seconds after needle withdrawal) and after pain (20 minutes after needle withdrawal) measures performed in experiment 1.

### Data processing and analysis

For each muscle, the 50 ms of the EMG signal preceding the stimulus artefact was visually checked for contamination by voluntary muscle activity. Responses were removed from further analysis if silence in the EMG signal was not maintained (<5 % of recordings discarded). The remaining responses in each condition were averaged and the peak-to-peak amplitude of the averaged MEP response was extracted (Signal 3, Cambridge Electronic Design, Cambridge, UK). At each conditioning stimulus intensity, SICI was determined by expressing the averaged MEP amplitude of the conditioned response in VL relative to the averaged MEP amplitude of the corresponding test response in the VL [33].

### Statistical analysis

Normality of the dependent variable distributions was checked using Shapiro-Wilk tests. As MEP amplitudes were not normally distributed, Friedman’s tests were used to analyse changes in corticospinal excitability over time. Where a significant time effect was observed, Wilcoxon signed-rank tests were used to compare MEP amplitudes measured during pain and after pain to the before pain condition. Bonferroni adjustments were used to control for multiple comparisons. Two-way ANOVAs with factors of time (before pain, during pain, after pain) and conditioning stimulus intensity (55 %, 65 %, 75 % of RMT) were used to analyse differences in SICI over time. One-way repeated measures ANOVAs were utilised to analyse differences in test MEP amplitude during the measurement of SICI. Where the assumption of sphericity was violated, Greenhouse-Geisser corrections were utilised. A Mann-Whitney *U* test was used to compare peak pain intensity after hypertonic and isotonic saline injection. The alpha level for all statistical tests was set to 0.05. Data are presented as mean and standard error of the mean (SEM).

## Results

### Experimental knee pain

Following hypertonic saline injection, the average (± SEM) duration of knee pain was 20 ± 1.4 minutes. Following isotonic saline injection, one participant reported knee pain that lasted <60 s after needle withdrawal. All other participants reported being pain free. The average (± SEM) peak knee pain intensity after hypertonic saline injection was 5.0 ± 0.5 cm, significantly higher (Mann-Whitney: Z = −3.565, *p* <0.001) than isotonic saline injection (0.6 ± 0.6 cm).

### MEP amplitude

**Fig. 1:**
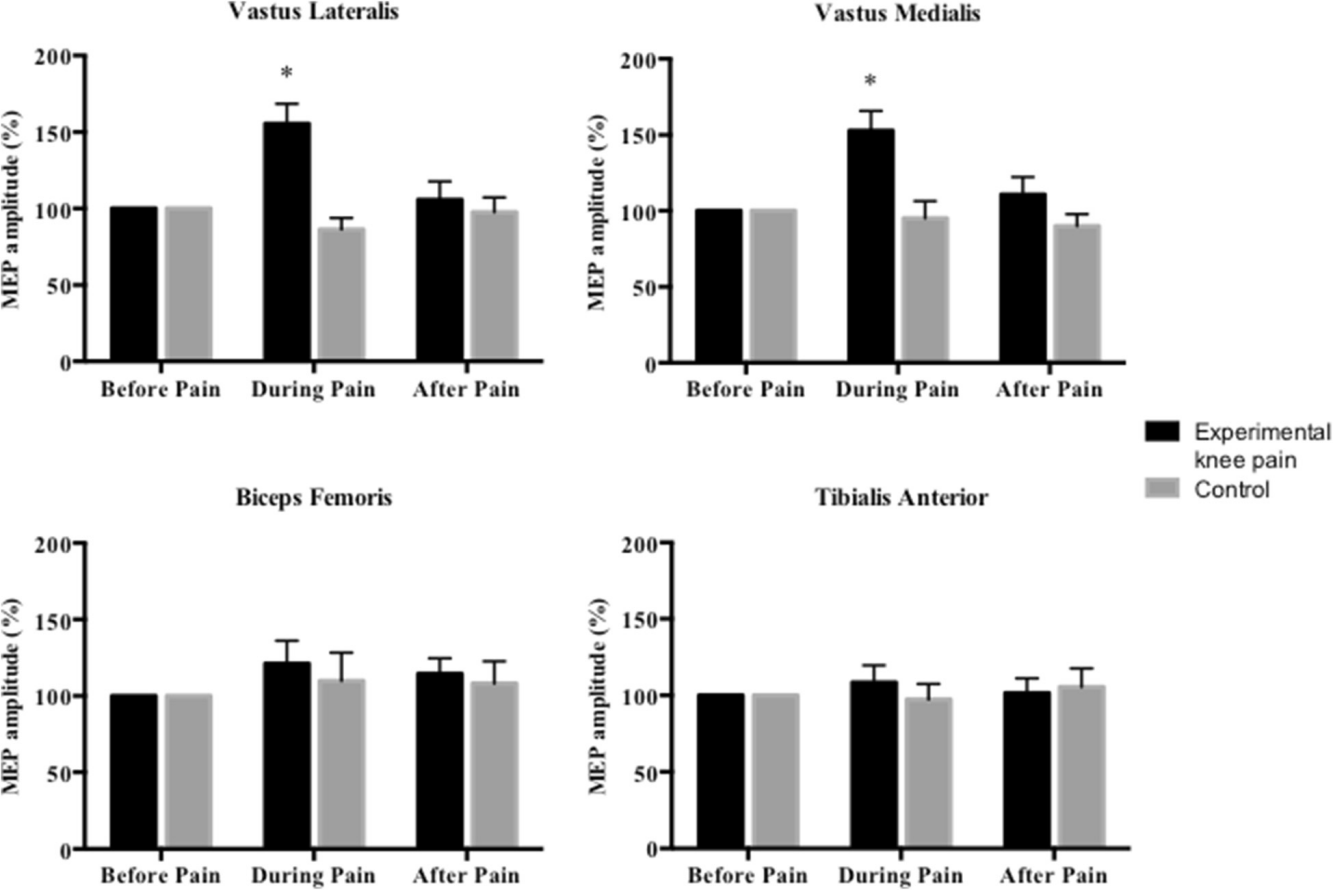
Mean (+ SEM) motor-evoked potential (MEP) amplitude measured from the right vastus lateralis, vastus medialis, biceps femoris and tibialis anterior muscles before and after the injection of hypertonic saline (n = 18) or isotonic saline (n = 8) into the right infrapatellar fat pad. Data are presented as a percentage of baseline (before pain). Significant difference compared with before pain (*, *p* <0.05)

There was a significant change in VL (Friedman: *χ*2 = 8.44, *p* = 0.01; Fig. 1) and VM (Friedman: *χ*2 = 10.33, *p* = 0.006; Fig. 1) MEP amplitude over time following hypertonic saline injection (experiment 1). Compared with the before pain condition, both VL (Z = −2.59; *p* = 0.02) and VM (Z = −2.59; *p* = 0.02) MEP amplitude increased significantly during pain. After pain, neither VL (Z = −0.85; *p* = 0.40) nor VM (Z = −0.24; *p* = 0.81) MEP amplitude were significantly different from before pain. There was no significant change in TA (Friedman: *χ*^2^ = 1.78; *p* = 0.41) or BF (Friedman: *χ*^2^ = 2.47; *p* = 0.21) MEP amplitude over time (Fig. 1).

Following control injection of isotonic saline (experiment 2) there was no significant change in VL (Friedman: *χ*^2^ = 4.75; *p* = 0.09), VM (Friedman: *χ*^2^ = 0.25; *p* = 0.88), TA (Friedman: *χ*^2^ = 0.25; *p* = 0.88) or BF MEP amplitude (Friedman: *χ*^2^ = 1.75; *p* = 0.42) over time (Fig. 1).

### Short-interval intracortical inhibition

**Fig. 2:**
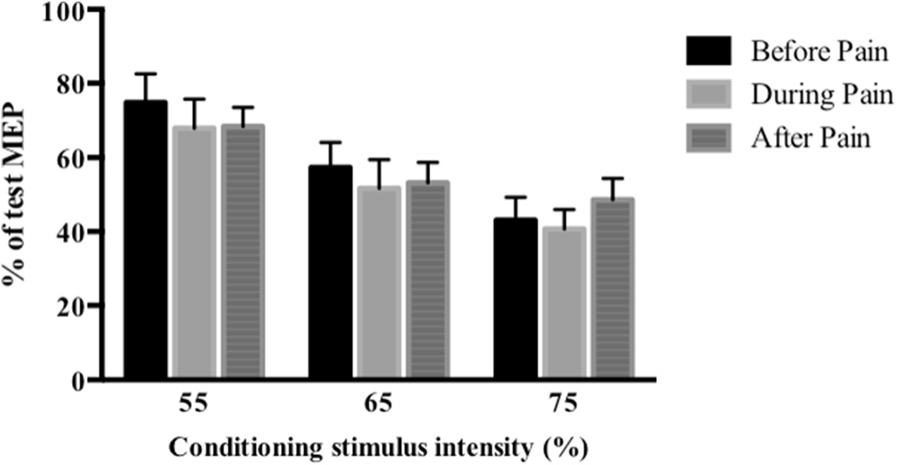
Mean (+ SEM, n = 18) short-interval intracortical inhibition (SICI) measured in the right vastus lateralis muscle before and after the injection of hypertonic saline into the right infrapatellar fat pad. Data are presented as the percentage of test motor-evoked potential (MEP) amplitude in each condition with conditioning stimulus intensities of 55 %, 65 % and 75 % of resting motor threshold

Following hypertonic saline injection there was no significant change in VL SICI over time (Fig. 2) (ANOVA: F = 0.59; *p* = 0.53) and no time by conditioning stimulus intensity interaction effect (ANOVA: F = 1.09; *p* = 0.37). As expected, there was a main effect for conditioning stimulus intensity (ANOVA: F = 14.42; *p* <0.001).

Following isotonic saline injection there was no significant change in VL SICI over time (ANOVA: F = 2.03; *p* = 0.17) and no time by conditioning stimulus intensity interaction effect (ANOVA: F = 1.07; *p* = 0.36). As expected, there was a main effect for conditioning stimulus intensity (ANOVA: F = 6.83; *p* = 0.03).

During the measurement of SICI, consistent test MEP amplitudes were maintained across all measurement points following both hypertonic injection (ANOVA: F = 1.66; *p* = 0.21) and isotonic injection (ANOVA: F = 0.25; *p* = 0.78).

## Discussion

The main finding of this study was the increased corticospinal excitability in both the VM and VL portions of the quadriceps muscle during experimental knee pain that was not found in a control condition. While unexpected, these observations are consistent with previous findings in individuals with chronic anterior knee pain [35] and ACL injury [36], where quadriceps corticospinal excitability was found to be increased compared with healthy control participants, or the uninjured limb. Furthermore, a recent study in patients with knee OA [37] demonstrated a positive linear relationship between joint pain intensity and quadriceps corticospinal excitability.

Interestingly, the observed increase in corticospinal excitability appears to be unique to the quadriceps muscle group, as BF and TA MEP amplitude did not change over time. This pattern is consistent with findings in individuals with chronic anterior knee pain, where VM and VL MEP amplitude were significantly increased compared with healthy controls, but no between-group difference was observed in MEP amplitude recorded from the extensor digitorum brevis [35].

Previous studies using the hypertonic saline model have shown significant decreases in quadriceps peak torque [23, 24], voluntary activation [24] and H-reflex amplitude [24] during knee pain. The observed decrease in H-reflex amplitude suggests that at least part of this inhibitory response occurs due to spinal reflex inhibition of quadriceps α-motoneurons. While H-reflex amplitude is influenced by factors independent of motoneuron excitability such as Ia afferent presynaptic inhibition, animal studies have shown that nociceptive input from the knee decreases, rather than increases Ia afferent presynaptic inhibition [38]. Thus, decreased H-reflex amplitude likely reflects postsynaptic inhibition of the quadriceps α-motoneuron pool. As corticospinal excitability is partly determined by α-motoneuron excitability, the present observations of an *increase* rather than a *decrease* in quadriceps MEP amplitude suggest that, despite spinal inhibition of quadriceps α-motoneurons, experimental knee pain leads to an increase in excitability elsewhere in the corticospinal pathway, either at a cortical or subcortical level.

The current study is the first to directly measure changes in motor cortex excitability during experimental knee pain using paired-pulse TMS. Despite a thorough investigation of SICI at three different conditioning stimulus intensities, none of the present results indicate that this pathway is altered by experimental knee pain. This finding is consistent with previous findings showing no difference in quadriceps SICI between healthy controls and individuals with knee OA [37]. Importantly, the lack of change in SICI does not exclude the possibility of altered motor cortex excitability contributing to the increase in quadriceps MEP amplitude during pain. There are various inhibitory and excitatory influences on corticospinal tract neurons in the motor cortex that may influence MEP amplitude. These include the neural circuitry involved in short and long-interval intracortical facilitation, long-interval intracortical inhibition, and interhemispheric inhibition [39]. Thus, it is possible that the observed increase in quadriceps MEP amplitude reflects an increase in motor cortex excitability via intracortical pathways independent of SICI [40]. Unfortunately, in the current study the limited time course of the experimentally induced pain (less than 10 minutes in some individuals) precluded the collection of other variables such as short and long-interval intracortical facilitation. It was decided to focus on SICI as this pathway may modify corticospinal drive during voluntary muscle contractions [41, 42] and previous research has shown that SICI is increased following acute muscle pain [28].

Finally, it is possible that the increase in quadriceps MEP amplitude may be explained by altered excitability in subcortical structures. In proximal lower limb muscles such as the quadriceps, a significant portion of the corticospinal input to the motoneuron pool is relayed via lumbar group II interneurons [43], which are thought to form part of the lumbar propriospinal system [44, 45]. Animal studies have shown that these interneurons receive strong excitatory input from knee joint afferents [46]. Thus, it is possible that the increase in quadriceps MEP amplitude is due to a joint nociceptor-mediated increase in the excitability of lumbar propriospinal pathways. In turn, this would greatly facilitate the portion of the descending MEP volley that is relayed via this pathway.

This is the first study to assess changes in corticospinal excitability of lower limb muscles in response to acute pain. The findings are different to those observed in the upper limb, where acute pain has generally been found to suppress MEP amplitude [27, 28]. Furthermore, in contrast to the current study, experimental pain in a hand muscle was shown to increase SICI [28]. It is unclear whether these differences are related to the source of pain (joint versus muscle), the location of pain (upper versus lower limb), or the different functional roles of the tested muscles (e.g. motor dexterity versus locomotion).

Regardless of the reasons for the difference in findings between the current study and previous work, the present observations provide further experimental support that the motor response to pain is more complex and variable than previously thought [47]. Rather than a uniform inhibitory response to pain that suppresses motor output globally or in the agonist muscle [48], pain appears to have different and at times competing effects (i.e. inhibition versus facilitation) at different levels of the motor system (e.g. cortex versus spinal cord) [47]. Furthermore, this response appears to depend on the muscle involved and/or the location of pain. Different parts of the body have distinct functional roles and hence, different motor adaptations to pain seem appropriate. For example, while uniform inhibition of spinal and cortical motor centres may typically be adaptive in response to acute hand pain, uniform motor inhibition may not always be appropriate in the lower limb, which is required for locomotion and escape behaviours.

A limitation of the current study is that we did not include a measure of quadriceps muscle strength and/or activation. As such, we cannot be sure that quadriceps AMI occurred during knee pain. However, as strong voluntary contractions are known to produce large and persistent changes in corticospinal excitability [49], including these measures would almost certainly have obscured our ability to observe changes in the dependent variables in response to knee pain. Importantly, previous studies using the same model of experimental knee pain have demonstrated an immediate decrease in quadriceps peak torque [23, 24], voluntary activation [24] and H-reflex amplitude [24] during pain. Finally, it should be noted that hypertonic saline injection produces transient knee pain that may not accurately mimic the pain experienced by clinical populations after acute knee injury, surgery or pathology. However, the infrapatellar fat pad is richly innervated by nociceptors [50] and is a clinically important source of pain in common knee joint pathologies such as patellofemoral syndrome [51], osteoarthritis [52] and rheumatoid arthritis [53]. Furthermore, the validity of our results is strengthened by previous findings of increased quadriceps corticospinal excitability following experimental knee joint effusion [54], in individuals with chronic anterior knee pain [35], ACL injury [36] and painful knee OA [37].

## Conclusions

This study demonstrated a significant increase in quadriceps corticospinal excitability during experimental knee pain and no change in SICI either during or after pain. The mechanisms underlying the increase in quadriceps corticospinal excitability during acute knee pain remain unclear and should be investigated in future research, along with their potential functional consequences.

## Abbreviations

ACL: anterior cruciate ligament
AMI: arthrogenic muscle inhibition
BF: biceps femoris
EMG: electromyography
MEP: motor-evoked potential
OA: osteoarthritis
RMT: resting motor threshold
SICI: short-interval intracortical inhibition
TA: tibialis anterior
TMS: transcranial magnetic stimulation
VAS: visual analogue scale
VL: vastus lateralis
VM: vastus medialis
